# Cerebralcare Granule® enhances memantine hydrochloride efficacy in APP/PS1 mice by ameliorating amyloid pathology and cognitive functions

**DOI:** 10.1186/s13020-021-00456-9

**Published:** 2021-06-28

**Authors:** Ou Qiao, Xinyu Zhang, Yi Zhang, Haixia Ji, Zhi Li, Xiaoying Han, Wenzhe Wang, Xia Li, Juan Wang, Changxiao Liu, Wenyuan Gao

**Affiliations:** 1 Tianjin Key Laboratory for Modern Drug Delivery and High-Efficiency, School of Pharmaceutical Science and Technology, Tianjin University, Weijin Road, Tianjin, 300072 China; 2 The State Key Laboratories of Pharmacodynamics and Pharmacokinetics, Tianjin, 300193 China

**Keywords:** Alzheimer’s disease, Cerebralcare Granule®, Memantine hydrochloride, Cognitive impairments, Synaptic plasticity, Aβ plaque accumulation, Complementary medicine

## Abstract

**Background:**

Alzheimer’s disease (AD) is a progressive neurodegenerative disease characterized by memory deficits and cognitive decline. Current drugs can only relieve symptoms, but cannot really cure AD. Cerebralcare Granule® (CG) is a Traditional Chinese medicine (TCM) containing a variety of biologically active compounds. In our previous studies, CG has shown a beneficial effect against memory impairment in mice caused by d-galactose. However, whether CG can be used as a complementary medicine for the treatment of AD remains unexplored. Here, we use a combination of CG and memantine hydrochloride (Mm) to treat Alzheimer-like pathology and investigate the effects and mechanisms in vivo.

**Methods:**

The histology of brain was examined with Hematoxylin–eosin (HE) staining, Golgi staining and Thioflavin S staining. ELISA was applied to assess the expression levels or activities of CAT, SOD, GSH-Px, MDA, alanine aminotransferase (ALT), aspartate aminotransferase (AST), alkaline phosphatase (ALP), total bilirubin (TBIL) in serum, as well as the levels of IL-6, IL-1β, and TNF-α in the mice brain. Western blotting was used to assess the expression of β-secretase (BACE1), amyloid precursor protein (APP), APPβ, APPα, synaptophysin (SYN), growth-associated protein 43 (GAP43), and postsynaptic density 95 (PSD95).

**Results:**

In the present study, the combination group (CG + Mm) significantly attenuated Alzheimer-like behavior without adverse effects in APP/PS1 mice, indicating its high degree of safety and efficacy after long-term treatment. CG + Mm reduced AD pathological biomarker Aβ plaque accumulation by inhibiting BACE1 and APP expression (*P* < 0.05 or *P* < 0.001). Besides, the combination group markedly inhibited the levels of IL-1β, IL-6, and TNF-α in hippocampus (*P* < 0.001), as well as activities of SOD, CAT, and GSH-Px in serum (*P* < 0.001). By contrast, the combination group improved synaptic plasticity by enhancing SYN, PSD95, and GAP43 expression.

**Conclusions:**

Taken together, these data supported the notion that CG combined with Mm might ameliorate the cognitive impairment through multiple pathways, suggesting that CG could play a role as complementary medicine to increase anti-AD effect of chemical drugs by reducing Aβ deposition, neuroinflammation, oxidative damage, and improving synaptic plasticity.

## Background

AD is the most common form of dementia affecting the elderly [1]. AD patients suffered progressive cognitive and functional deficits, which resulted in a heavy burden to patients, families, and the public health system [2]. In 2019, AD affected more than 50 million people globally, which is expected to reach 152 million by 2050. In addition, the current annual cost of AD worldwide is $1 trillion, which is estimated to double by 2030 [3]. Rising prevalence and mortality, and the lack of effective treatments, have resulted in huge costs to society. Currently, cholinesterase inhibitors (i.e., donepezil) and Nmethyl-d-aspartate (NMDA) receptor antagonists (i.e., memantine) are approved by FDA for the treatment of AD. However, these drugs can only moderately relieve symptoms and cannot really cure AD. Therefore, we need to find new treatment options to slow down the progression of AD.

Accumulation of Aβ is considered to be one of the main pathogenic features of AD, which causes a series of neuronal damage [4]. Aβ plaques affect synaptic morphology and function by disrupting the synaptic signaling pathway, leading to memory loss and behavioral changes [5]. Moreover, Aβ oligomers deposited extracellular can also activate microglia and subsequently release inflammatory cytokines, the amount of APP will increase due to the large release of these factors. As the amount of APP increases, the production of Aβ becomes higher [6, 7]. Severe oxidative stress and neuroinflammation, loss of synaptic connections in specific brain regions, cumulative emergence of intracellular tau pathology, and accumulation of extracellular amyloid Aβ plaques form a complex neurodegeneration [8, 9]. Therefore, oligomeric Aβ plaques are considered to be one of the main pathological factors of AD.

**Fig. 1:**
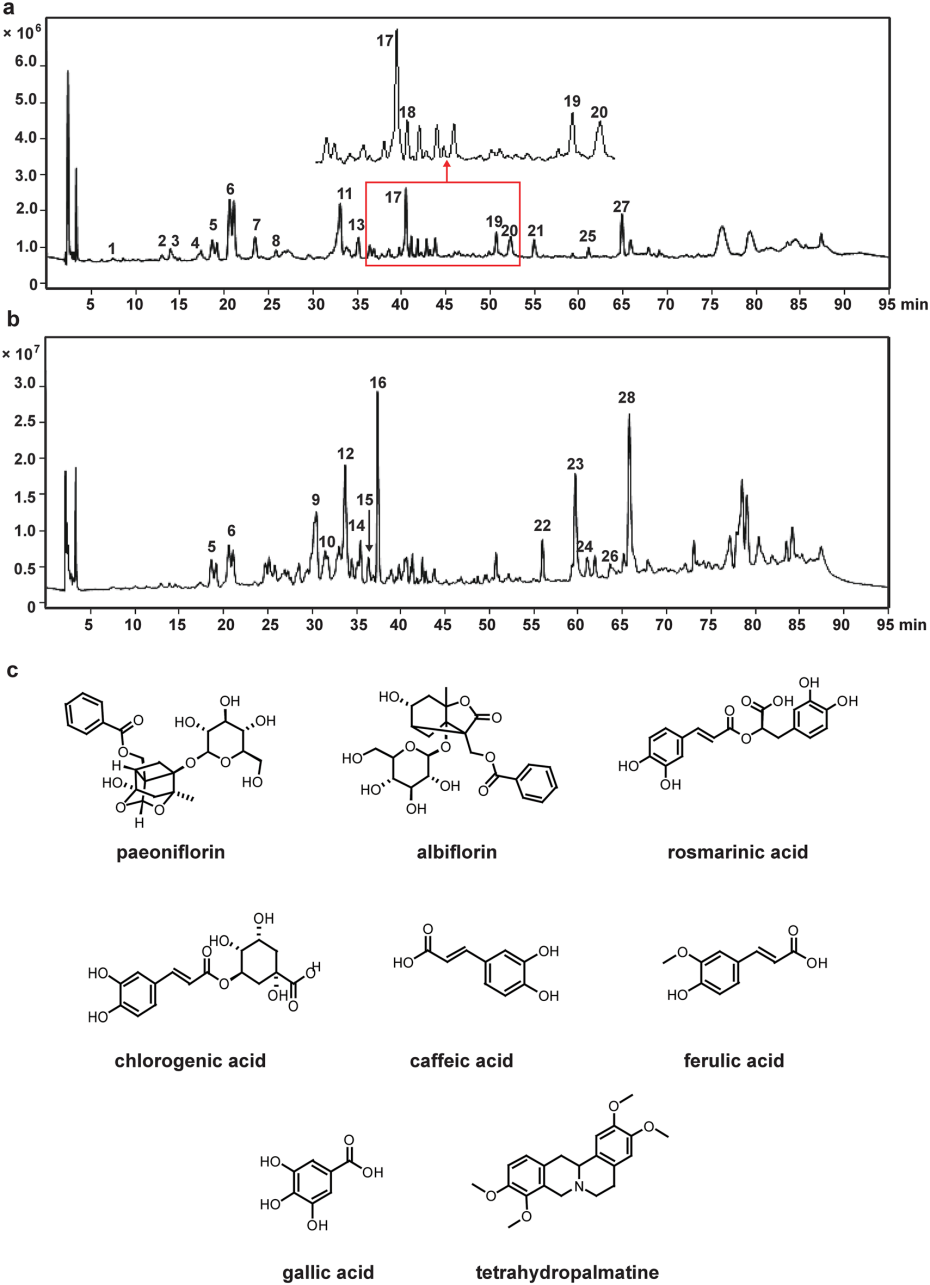
The chemical composition analysis of CG. **a** Total ion chromatogram of GC in negative ion mode; **b** Total ion chromatogram of CG in positive ion mode. **c** The chemical structures of eight active ingredients in CG

CG (Tianjin Tasly Pharmaceutical Co., Ltd, Tianjin, China) was approved by the National Medical Products Administration (NMPA) in 1996 for treatment of headache and dizziness associated with cerebrovascular diseases. It is consists of 11 herbs, including Angelicae Sinensis Radix, Paeoniae Radix Alba, Chuanxiong Rhizoma, Rehmanniae Radix Praeparata, Uncariae Ramulus Cum Uncis, Spatholobi Caulis, Corydalis Rhizoma, Prunellae Spica, Margarita, Cassiae Semen, and Asari Radix et Rhizoma. These 11 herbs contain a large number of chemical components. Some of these ingredients are key to the treatment of diseases in TCM and are the main sources of new drug lead compounds [10]. For example, paeoniflorin, albiflorin, rosmarinic acid, chlorogenic acid, tetrahydropalmatine, caffeic acid, gallic acid, and ferulic acid (Fig. 1c) are known to improve learning and memory deficits, attenuate neurotoxicity, and anti-oxidant damage [11–15]. Previous studies have shown that the ingredients mentioned above can be detected in blood of mice after oral administration of CG [16, 17]. Due to the complicated mechanisms of TCM, these ingredients were usually used as part of a combination therapy rather than monotherapy [18]. Studies have shown that as a supplementary treatment of modern medicine, TCM improved the therapeutic effect of modern medicine. For example, after intraperitoneal injection of pentobarbital sodium, rats were given He jie Zhitong prescription by gavage, the sleep latency of rats was significantly decreased, and the sleep time was prolonged, suggesting that Hejie Zhitong prescription exerted a synergistic effect with pentobarbital sodium [19]. Shufeng Jiedu Capsule enhanced doxorubicin therapeutic efficacy in hepatocellular carcinoma by inhibiting migration and invasion [20]. Fuzheng Kang Ai decoction combined with erlotinib enhances the effect of lung cancer treatment [21]. Xiaoaiping injection enhanced paclitaxel efficacy in ovarian cancer proliferation by inhibiting pregnane X receptor [22]. Huyang Yangkun Formula could enhance the therapeutic effect of embryonic stem cells on premature ovarian failure mice. This combined therapy could promote the development of mice follicles and inhibit the expression of the TGF-β1/TAK1 pathway [23]. These results suggest that the combination therapy of TCM and western medicine is feasible in the treatment of various diseases. However, there were no studies on attenuation Alzheimer-like pathology of combination therapy of CG and other chemotherapy drugs.

Mm is a moderate-affinity, uncompetitive NMDA receptor antagonist. It is used to improve cognitive and behavioral disorders in moderate to severe AD patients [24, 25]. Studies have shown that Mm can reduce Aβ levels in 5xFAD mice [25], but its mechanism is still unclear. Within Mm therapeutic dose range, this property can be further enhanced by combining other drugs to benefit the treatment of AD. To illustrate whether CG can enhance the therapeutic effect of Mm on AD, the APP/PS1 mice model was used to solve the puzzle. As a result, the combination of CG and Mm had a stronger anti-AD effect via reducing Aβ plaque deposition than the single treatment. Meanwhile, the expressions of APP, APPβ, APPα, BACE1, PSD95, SYN, and GAP43 have correspondingly changed, which may be the reason for the synergistic anti-Alzheimer’s efficacy.

Our work provides a comprehensive explanation for the effect of CG combined with Mm against AD in terms of reducing Aβ deposition by inhibiting BACE1 expression, decreasing APPβ and APP levels, which indicates that CG and Mm has a synergistic effect on anti-Aβ production. Moreover, the combination therapy also has significant anti-inflammatory, anti-oxidant damage, and maintaining synaptic connection effects. The present study providing a new idea combination therapy of AD for the combination treatment of Chinese medicine and Chemical drugs.

## Methods and materials

### Chemicals and antibodies

CG was obtained from Tianjin Tasly Pharmaceutical Co., Ltd (Tianjin, China). Mm was obtained from H. Lundbeck A/S (Denmark). MDA, CAT, GSH-Px, SOD, IL-6, IL-1β, and TNF-α ELISA kits were obtained from Shanghai FANKEL Industrial Co., Ltd (Shanghai, China). ALT, AST, ALP, and TBIL assay kits were purchased from Nanjing Jiancheng Bioengineering Research Institute (Nanjing, China). The following antibodies Aβ, APP, APPα, APPβ, SYN, PSD95, GAP43, BACE 1, β-actin, and GAPDH were obtained from Boster Biological Engineering Co., Ltd (Wuhan, China).

### HPLC-QQQ-MS/MS analysis condition

CG (1.0 g) was ultrasonically extracted with 10 mL of 70% methanol for 30 min (weight: volume = 1:10). The weight loss was made up with 70% methanol. The extract was centrifuged at 4000 rpm for 10 min and the supernatant was filtered by a 0.22 mm filter before analysis. The chemical constituents of CG were analyzed by Agilent 1200 series HPLC system (Agilent Technologies, USA) connected with the electrospray ionization tandem triple quadrupole mass spectrometry (ESI-QQQ-MS, Bruker Daltonics Inc., USA). Chromatographic separation was performed on a Kromasil 100-5-C18 column (4.6 mm × 250 mm, 5 μm) using two mobile phases: ultrapure water with 0.1% formic acid (A) and acetonitrile (B). The gradient elution program was shown as follows: 0–5 min, 5–10% B; 5–30 min, 10–25% B; 30–58 min, 25–65% B; 58–78 min, 68–88% B; 78–85 min, 88–93% B; 85–95 min, 93–5% B. The flow rate: 1 mL/min, the column temperature was 28 °C, and the injection volume: 10 μL. The MS analysis was worked using full scan mode and the mass range was recorded from m/z 100 to 1000 both in positive mode and negative mode. The MS conditions were as follows: drying gas (N_2_) flow rate: 8.0 L/min; the drying gas temperature was set to 320 °C; nebulizer pressure: 40 psig; capillary voltage: 3000 V; fragmentor voltage: 135 V. Bruker Compass Data Analysis software Version 4.3 (Bruker Daltonics GmbH, Bremen, Germany) was used for data acquisition and analysis.

### Animals and drug administration

Six month old male C57BL/6J wild-type mice and APP/PS1 mice were provided by the SPF (Beijing) Biotechnology Co., Ltd in China (license no. SCXK (Jing) 2019–0010). All mice were adaptively reared for one week before the initiation of experimentation. The mice were kept in standard cages at 23 ± 2 °C under 12 h/12 h light/dark conditions and a humidity of 40 ± 5%, and they were provided with mice food and water ad libitum. All experimental protocols were conducted according to guidelines of the Institutional Animal Care and Use Committee of Institute of Radiation Medicine Chinese Academy of Medical Sciences (Tianjin, China). The mice were randomly divided into five groups (10 mice per group): Wild type group (WT, saline), APP/PS1 mice group (Tg, saline), CG group (2.46 g/kg, based on clinical dosage), Mm group (Mm, 5 mg/kg, according to the Zhang JH et al. [26]), CG combined with Mm group (CG + Mm, 2.46 mg/kg, 5 mg/kg, respectively). All drugs were dissolved in 0.9% physiological saline. The treatments were administered intragastricly once daily for 4 weeks. After treatment, The Morris water maze test and Open-field test were used to evaluate cognitive ability and behavioral changes of mice.

### Morris water maze (MWM) test

**Fig. 2:**
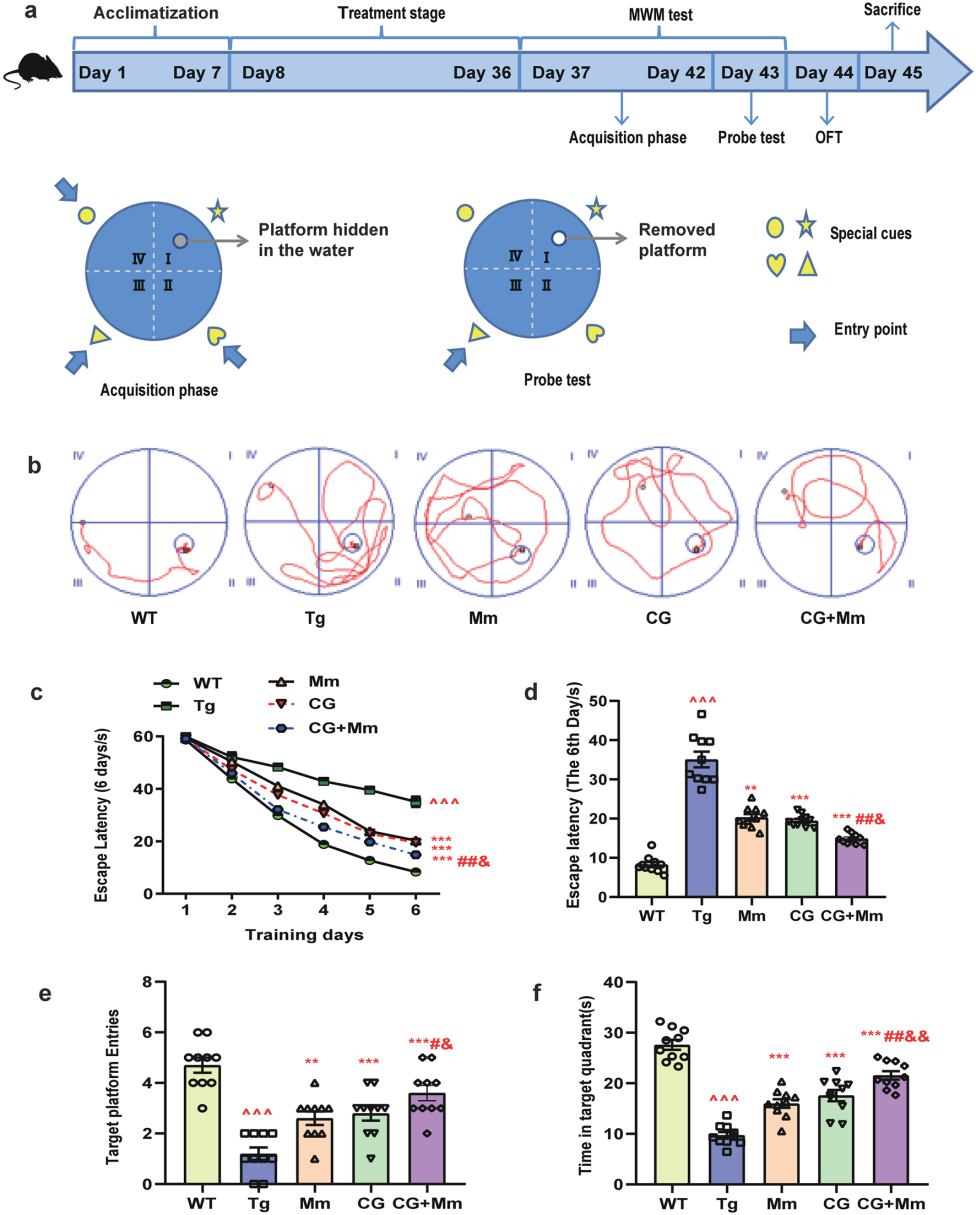
The scheme of experiment and MWM test. **a** The scheme of experiment to determine the effect of CG on APP/PS1 memory loss. **b**–**f** MWM test was conducted on APP/PS1 mice. **b** The representative traces on the last day during acquisition phase. **c** Escape latency trend for six consecutive days. **d** Escape latency on the sixth day. **e** Times of passing through platform. **f** Time in the target quadrant. ^ *P* < 0.05, ^^ *P* < 0.01, ^^^ *P* < 0.01 vs. WT group; * *P* < 0.05, ** *P* < 0.01, *** *P* < 0.01 vs. Tg group; # *P* < 0.05, ## *P* < 0.001, ### *P* < 0.001, vs. Mm group, & *P* < 0.05, && *P* < 0.001, &&& *P* < 0.001, vs. CG group (n = 10)

The MWM video tracking analysis system (Beijing Zhishuduobao Biological Technology Co., Ltd. China) was used to assess the learning and memory ability of mice in spatial position and orientation after treatment. The water depth of the MWM system was 20 cm, the diameter was 120 cm, and the water temperature was (23 ± 2) °C. The platform was placed in the second quadrant, submerged about one cm below the water surface. In brief, Spatial training to find the target platform hidden in the water and was performed consecutively for six days. During the acquisition phase (days 1–6), animals that failed to find the platform within 60 s were manually guided to the platform and given 20 s to stay. The escape latency (EL) for finding the submerged platform was regarded as 60 s. Twenty-four hours after the final spatial training, the probe test was performed by removing the platform and giving each mice to swim freely for 60 s. The number of times they crossed platform and the time in the second quadrant were automatically recorded by the MWM video tracking system. The experimental scheme as shown in Fig. 2a.

### Open field test (OFT)

The experimental device was a square Plexiglas box, the bottom plate was divided into 25 squares by white lines, and the wall was a black color. The mice were placed in the same position in the box, and the total distance traveled of mice within 5 min was recorded by a video tracking system at the top of the experimental platform. The field instrument was swabbed with 75% alcohol to avoid leaving an abnormal odor after each mice had finished recording. The following observations were made: number of square crossings, exercise time and the trajectories of the mice.

### Hematoxylin–eosin (HE) staining

The brain tissues were immersed in 4% paraformaldehyde solution and fixed at room temperature for 24 h, then dehydrated according to standard procedures, embedded, sliced 10 μm, baked slices, stained with HE and sealed slices with gum, etc. The pathological changes of each group of brain tissues were observed under a light microscope.

### Golgi staining for dendritic spines

After anesthesia, three brains of mice were collected and fixed in 4% paraformaldehyde solution for 24 h. After fixation, the brain tissues were stained in Golgi staining solution for 14 days without light. After soaking for 48 h, the brain tissues were transferred to a new Golgi staining solution. Next, the liquid was changed every three days. Brain tissues were taken out and placed in 15% sucrose solution for dehydration at 4 °C for one day, and brain tissues were taken out and placed in 30% sucrose solution for dehydration at 4 °C for two days. The brain tissues were taken out and washed successively with distilled water for 60 s, concentrated ammonia for 45 min, distilled water for one min, fixing solution for 45 min and distilled water for one min. The brain tissues were placed in 30% sucrose solution for dehydration at 4 °C and avoided light for three days. After dehydration, slices of 100 μm thickness were cut with a frozen slicer, and the slices were sealed with glycerin gelatin. The sections were observed under oil microscope, and the sections with the same position and field of view were taken photos. The Image processing software Image J was used to calculate the density of dendritic branches and dendritic spines in each section.

### Thioflavin S staining

The brain tissue was placed in paraformaldehyde and sectioned 24 h later with paraffin embedding. Thioflavine-S was immersed in 0.125% thioflavine-S solution for 8 min in darkness. Wash the tablets with PBS for three times, five min each time, and then seal the tablets with sealing tablets. The deposition of Aβ in the hippocampus of each group of mice was analyzed under microscope.

### Biochemical assays

According to the manufacturer’s protocol, the concentrations of IL-10, TNF-α, and IL-6 in the hippocampus were measured by ELISA Kit (Nanjing Jiancheng Bioengineering Research Institute, Nanjing, China). 1 g of hippocampal tissue was washed with cold 0.9% physiological saline and wiped with filter paper. Tissue samples were placed in 0.1 mol phosphate buffered saline (10 mL, pH 7.4) at 4 °C and treated with a tissue crusher. The homogenate was centrifuged at 4500 rpm for 10 min. The supernatant was collected and used to evaluate tissue biochemical parameters.

The blood samples of mice were collected and centrifuged (4500 rpm for 10 min) to isolate the serum, which was frozen at − 20 °C prior to investigation. The changes of CAT, SOD, GSH-Px, ALT, AST, ALP, and TBIL in serum were detected by ELISA kit (Nanjing Jiancheng Bioengineer Institute, Nanjing, China) according to their manufacture’s instruction.

### Western blotting

The protein was isolated from the hippocampus of the brain according to the instructions of the protein extraction kit (Shanghai FANKEL Industrial Co., Ltd. Shanghai, China). The protein concentration was determined by BCA protein assay kit (Shanghai FANKEL Industrial Co., Ltd. Shanghai, China). 50 µg protein per sample was separated by SDS-PAGE and transferred to the PVDF membrane. Membranes were blocked in 5% non-fat milk for 1 h. After blocking, the membranes were incubated with primary antibody at 4 °C overnight. Antibodies as follows: anti-SYN (1: 1000, Abcam Technology, UK), anti-PSD95 (1: 2000, Abcam Technology, UK), anti-GAP43 (1: 1000, Abcam Technology, UK), anti-APP (1: 1000, Abcam Technology, UK), anti-BACE1 (1: 1000, Millipore Technology, USA), anti-APPα (1: 1000, Wuhan Servicebio Technology Co., LTD, China), anti-APPβ (1: 1000, Wuhan Servicebio Technology Co., LTD, China), anti-β-actin (1: 1000, Abcam Technology, UK), anti-GAPDH (1: 1000, Abcam Technology, UK). Membranes were rinsed with TBS-T three times, 5 min per each time. Then, membranes were incubated with horseradish peroxidase onjugated anti-rabbit or anti-mouse secondary antibodies at room temperature for 2 h. Finally, protein bands were visualized with the chemiluminescence reagents provided with the ECL kit (Bioworld, USA), and quantitation of proteins was determined by densitometric analysis using ImageJ software (Bio-Rad, Hercules, USA).

### Statistical analysis

All values are expressed as mean ± SEM. The data were analyzed by one-way analysis of variance (ANOVA) followed by Turkeys test for post hoc analysis. Tests were performed on SPSS 20.0 system (Chicago, IL); *P* ≤ 0.05 was considered to be statistically significant. ^ *P* < 0.05, ^^ *P* < 0.01, ^^^ *P* < 0.01 vs. WT group; * *P* < 0.05, ** *P* < 0.01, *** *P* < 0.01 vs. Tg group; # *P* < 0.05, ## *P* < 0.001, ### *P* < 0.001, vs. Mm group, & *P* < 0.05, && *P* < 0.001, &&& *P* < 0.001, vs. CG group.

## Results

### Chemical composition analysis of CG by HPLC-QQQ-MS

The HPLC-QQQ-MS/MS method was established to characterize the chemical constituents of CG. The MS total ion current profiles of CG were presented in the positive and negative mode (Fig. 1a, b). A total of 28 compounds (Table 1) were identified by comparing the retention time and MS fragmentation behaviors with literature information [27–31]. Most of these compounds have been reported as the main bioactive components of each individual herb.

**Table 1:** Chromatographic and mass data of the 28 compounds of CG analyzed by HPLC-QQQ-MS/MS

No	*t*_R_ (min)	Identified compound	Elemental composition	Theoretical m/z	Experimental m/z	Proposed ions	Common fragment ions/(m/z)	Plant source
1	6.19	Gallic acid	C_7_H_6_O_5_	169.0142	169.3922	[M−H]^−^	125.02, 107.02, 81.03	D
2	13.08	Chlorogenic acid	C_16_H_18_O_9_	353.0878	353.0875	[M−H]^−^	191.05, 179.03, 135.04	A, D, E, F, G
3	14.06	Isochlorogenic acid	C_16_H_18_O_9_	353.0878	353.0844	[M−H]^−^	–	E, F, G
4	17.37	Caffeic acid	C_9_H_8_O_4_	179.0350	179.0219	[M−H]^−^	135.04	B, E, G,
5	18.70	Albiflorin	C_23_H_28_O_11_	525.1614	525.1601	[M+COOH]^−^, [M+Na]^+^	525.16, 479.16, 327.11, 381.04, 341.15	D
6	20.47	Paeoniflorin	C23H28O11	525.1614	525.1545	[M+COOH]^−^, [M+Na]^+^	525.15, 479.16, 327.11 -	D
7	23.49	Ethyl gallate	C_9_H_10_O_5_	197.0455	197.0324	[M−H]^−^	169.12	D
8	25.90	Ferulic acid	C_10_H_10_O_4_	193.0506	193.0542	[M−H]^−^	149.06, 134.03	A, B
9	30.38	Tetrahydropalmatine	C_21_H_25_NO_4_	356.1856	356.1794	[M+H]^+^	341.16, 192.09, 165.08	I
10	31.57	Isocorynoxeine	C_22_H_26_NO_4_	369.1935	369.1836	[M+H]^+^	351.17, 319.14,187.13	E
11	33.13	Rosmarinic acid	C_18_H_16_O_8_	359.0772	359.0543	[M−H]^−^	197.04, 135.04, 161.02	G
12	33.66	Corydaline	C_22_H_27_NO_4_	370.2013	370.1958	[M+H]^+^	355.18, 340.16	I
13	34.75	Rubrofusarin-gentiobioside	C_27_H_32_O_15_	595.1668	595.1537	[M−H]^−^	273.08, 271.06, 256.04	H
14	35.45	Palmatine	C_21_H_22_NO_4_	352.1543	352.1592	M^+^	336.13, 321.10, 294.11,	I
15	36.43	Berberine	C_20_H_17_NO_4_	335.1152	335.1128	M^+^	321.10, 320.09, 304.10	I
16	37.41	Dehydrocorydaline	C_22_H_24_NO_4_	366.1700	366.1639	M^+^	351.15, 336.15, 321.10	I
17	40.50	Cassiaside C	C_27_H_32_O_15_	641.1723	641.1711	[M+COOH]^−^	641.21	H
18	41.07	Physcion-glucoside	C_22_H_22_O_10_	445.1140	445.1125	[M−H]^−^	283.06, 268.06, 212.04	H
19	50.85	aurantio-obtusin	C_17_H_14_O_7_	329.0667	329.0661	[M−H]^−^	314.04, 285.04, 299.02,	H
20	52.25	Rhein	C_15_H_8_O_6_	283.0248	283.0233	[M−H]^−^	239.03, 211.02	H
21	54.99	Physcion	C_16_H_12_O_5_	283.0612	283.0649	[M−H]^−^	268.03, 240.04, 239.04	H
22	56.05	chryso-obtusin	C_19_H_18_O_7_	357.0980	357.0982	[M−H]^−^	342.07, 312.03, 284.03	H
23	59.78	Senkyunolide A	C_12_H_16_O_2_	193.1223	193.1211	[M+H]^+^	173.10, 145.10, 135.05	A, B
24	60.97	Butylphthalide	C_12_H_14_O_2_	191.1067	191.1069	[M+H]^+^	173.10, 145.10, 135.05	A, B
25	61.11	Obtusifolin	C_16_H_12_O_5_	283.0612	283.0610	[M−H]^−^	268.03, 240.04	H
26	63.64	Z-Ligustilide	C_12_H_14_O_2_	191.1067	191.1.65	[M+H]^+^	173.10, 145.10, 135.04	A, B
27	64.83	Emodin	C_15_H_10_O_5_	269.0455	269.0451	[M−H]^−^	241.05, 225.05	H
28	65.75	E-Ligustilide	C_12_H_14_O_2_	191.1067	191.0834	[M+H]^+^	173.10, 145.10, 135.04	A, B

A: Angelica Sinensis Radix; B: Chuanxiong Rhizoma; C: Radix Rehmanniae Preparata; D: Radix Paeoniae Alba; E: Ramulus Uncariae Cum Uncis; F: Caulis Spatholobi; G: Spica Prunellae; H: Semen Cassiae; I: Rhizoma Corydalis Yanhusuo

### CG + Mm rescued cognitive deficits in AD mice

After treatment, we evaluated the spatial memory and autonomous exploration ability of AD mice by MWM and OFT. In the MWM test, compared with WT group, the Tg group showed an obviously memory impairment with longer EL (*P* < 0.001, Fig. 2c, d). Moreover, the mice in Tg group crossed the platform and target quadrant (the quadrant of the hidden platform) less frequently than WT mice (*P* < 0.001, Fig. 2e, f). After treatment, the trajectories of mice (CG, Mm, and CG + Mm group) tended to be normal (Fig. 2b), with swimming range primarily focusing on the second quadrant of the hidden platform. The EL was also significantly reduced by CG + Mm treatment (*P* < 0.001, Fig. 2c, d). Meanwhile, target quadrant residence time and times of crossing through target platform of APP/PS1 mice treated by CG + Mm were significantly increased (*P* < 0.001, Fig. 2e, f).

**Fig. 3:**
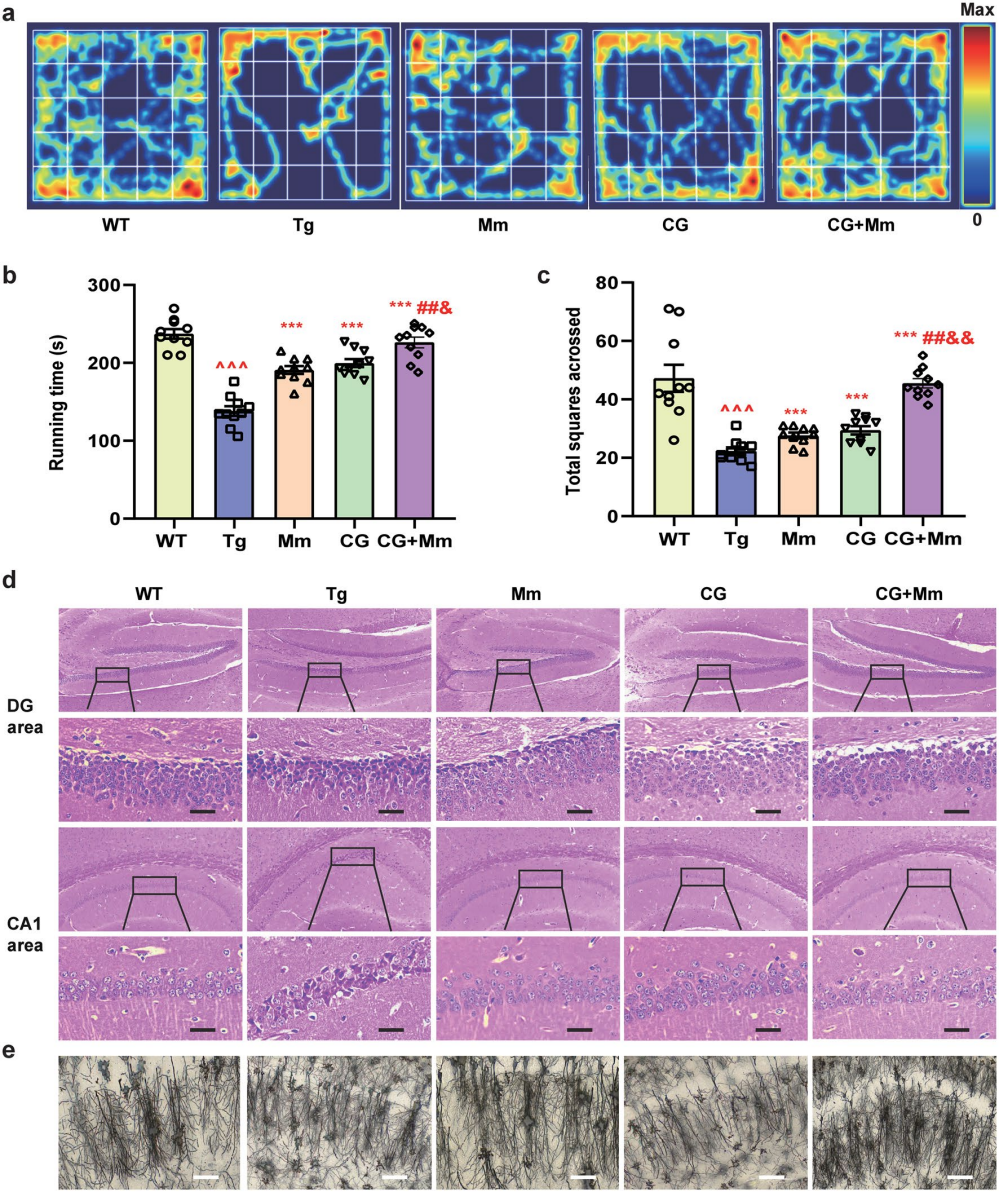
OFT and CG alleviated neuronal damage and improves synaptic function in mice brain. **a**–**c** OFT was conducted on APP/PS1 mice (n = 10). **a** The representative record of motion trajectories showed the locomotor path of all groups of mice. **b** Runnning time. **c** Total squares acrossed. **d** HE-stained images of hippocampal CA1 and DG area in APP/PS1. Scale bar = 200 μm. In APP/PS1 mice, the damaged neurons were deeply stained, and the neuron bodies were atrophied and appeared triangular. Cells are arranged indisorder with a slightly changed cell polarity. By contrast, treatment with CG, Mm, and CG + Mm significantly inhibited the histopathological damage. **e** Golgi staining of the hippocampus, Scale bar = 10 μm. ^ *P* < 0.05, ^^ *P* < 0.01, ^^^ *P* < 0.01 vs. WT group; * *P* < 0.05, ** *P* < 0.01, *** *P* < 0.01 vs. Tg group; # *P* < 0.05, ## *P* < 0.001, ### *P* < 0.001, vs. Mm group, & *P* < 0.05, && *P* < 0.001, &&& *P* < 0.001, vs. CG group (n = 10)

OFT was used to observe various behaviors of mice after being released into an open environment, reflecting the neuropsychological activities of experimental animals. As consequence, there were significant differences in the total running times and the total squares acrossed (Fig. 3a–c), indicating that the activity and exploration ability of the mice treated by CG + Mm were significantly improved. Above all, the CG + Mm significantly prolonged the time in the target quadrant and running time, increased times of total squares acrossed, and reduce the EL of mice (Figs. 2c–f; 3b, c). The results suggested that CG + Mm can significantly improve the learning and memory abilities and autonomous activities of APP/PS1 mice.

### CG + Mm rescues pathological changes in AD mice

HE staining and Golgi staining (Fig. 3d, e) was conducted to evaluate the effects of CG + Mm on neuronal loss in AD mice brain. In the mice of Tg group, the cells in the hippocampus were arranged disorderly, and some cells presented the characteristics of neurodegeneration, with darkly stained, and exhibited triangulated and shrunken neuronal bodies. The morphology of neurons in the hippocampus of mice in the CG, Mm, and CG + Mm group were improved to a certain extent, and the morphological characteristics of neurons were consistent, the cells were orderly and the cell structure was clear.

### CG + Mm improves synaptic function in AD mice

**Fig. 4:**
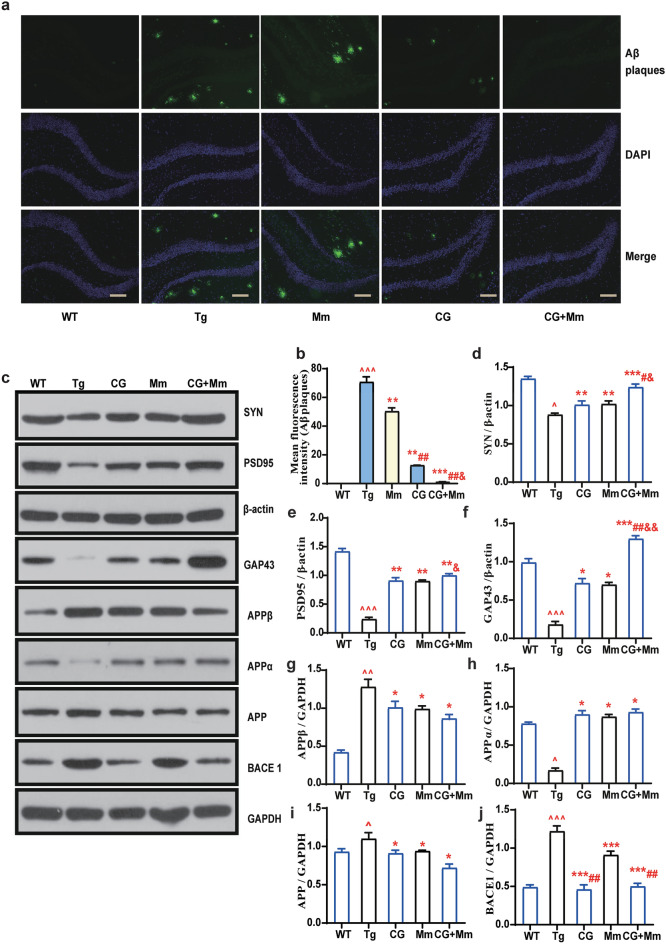
CG enhanced the effects of Mm on reducing Aβ plaque deposition In the hippocampus. **a** Immunofluorescence for Aβ in the hippocampus of the APP/PS1 mice. Scale bar = 100 μm. **b** Mean fluorescence intensity of Aβ plaques. **c** The protein levels of SYN, PSD95, GAP43, APPβ, APPα, APP, and BACE1 were measured by Western blotting in the hippocampus of the different groups (n = 10). **d**–**j** Quantitative analysis of SYN, PSD95, GAP43, APPβ, APPα, APP, and BACE1 expression, respectively. ^ *P* < 0.05, ^^ *P* < 0.01, ^^^ *P* < 0.01 vs. WT group; * *P* < 0.05, ** *P* < 0.01, *** *P* < 0.01 vs. Tg group; # *P* < 0.05, ## *P* < 0.001, ### *P* < 0.001, vs. Mm group, & *P* < 0.05, && *P* < 0.001, &&& *P* < 0.001, vs. CG group (n = 10)

The number of dendritic spines and the development of dendrites are closely related to the synaptic function of AD mice [32]. Here, we used Golgi staining to assess the effects of CG + Mm on dendritic morphology. As a result, Fig. 3e, the hippocampus of APP/PS1 mice in Tg group showed a lower density of spines, and this reduction could be rescued by CG, Mm, CG + Mm. We next quantified the expression of synapse-related proteins in the hippocampus of AD mice. Compared with the mice in Tg group, the expression of PSD95, SYN, and GAP43 in APP/PS1 mice treated with CG + Mm increased significantly (*P* < 0.01, *P* < 0.001, Fig. 4c). Moreover, the effect of CG combined with Mm was better than that of Mm or CG alone. This suggests that CG combined with Mm to improve synaptic function may be achieved by up-regulating the expression of PSD95, SYN, and GAP43 to regulate synaptic plasticity.

### CG enhanced the effects of Mm on reducing Aβ plaque deposition in AD mice

The Aβ has been proposed to play a key role in pathological progress [33], which accumulation and plaque deposition may induce dendritic and axonal atrophy, synaptic failure, and neuronal death [34, 35]. To confirm whether CG enhanced Mm alleviated cognitive impairment in AD mice by reducing Aβ plaque deposition, the brain sections were stained with thioflavin S for determination of Aβ plaques. Our study demonstrated that CG could lower Aβ deposition in the hippocampus compared to the Tg group and this treatment effect is stronger than that of Mm, as shown in Fig. 4a. In this study, Mm showed a weak positive effect on reducing Aβ deposition. By contrast, in the combined treatment group, this treatment has the strongest effect. The levels of BACE1, APP, APPα and APPβ in the hippocampus of AD mice were further confirmed with a Western blotting analysis. Similar to the immunofluorescence staining, APP levels in hippocampus were significantly reduced in the CG + Mm group (*P* < 0.05, Fig. 4i), as well as levels of APPβ (*P* < 0.05, Fig. 4g), BACE1 (*P* < 0.001, Fig. 4j). Conversely, the level of APPα increased significantly in CG + Mm group (*P* < 0.05, Fig. 4h). The CG group and the Mm group showed no significant difference except BACE1, which implies that CG + Mm has the strongest effect on inhibiting the expression of BACE1. In this study, we found that CG can enhance the effect of Mm in reducing Aβ deposition by down-regulating the expression of BACE1, APP and APPβ. The pathological changes of AD were significantly reversed.

### Effects of CG + Mm on neuroinflammation and oxidative stress in the brain and serum of AD mice

**Fig. 5:**
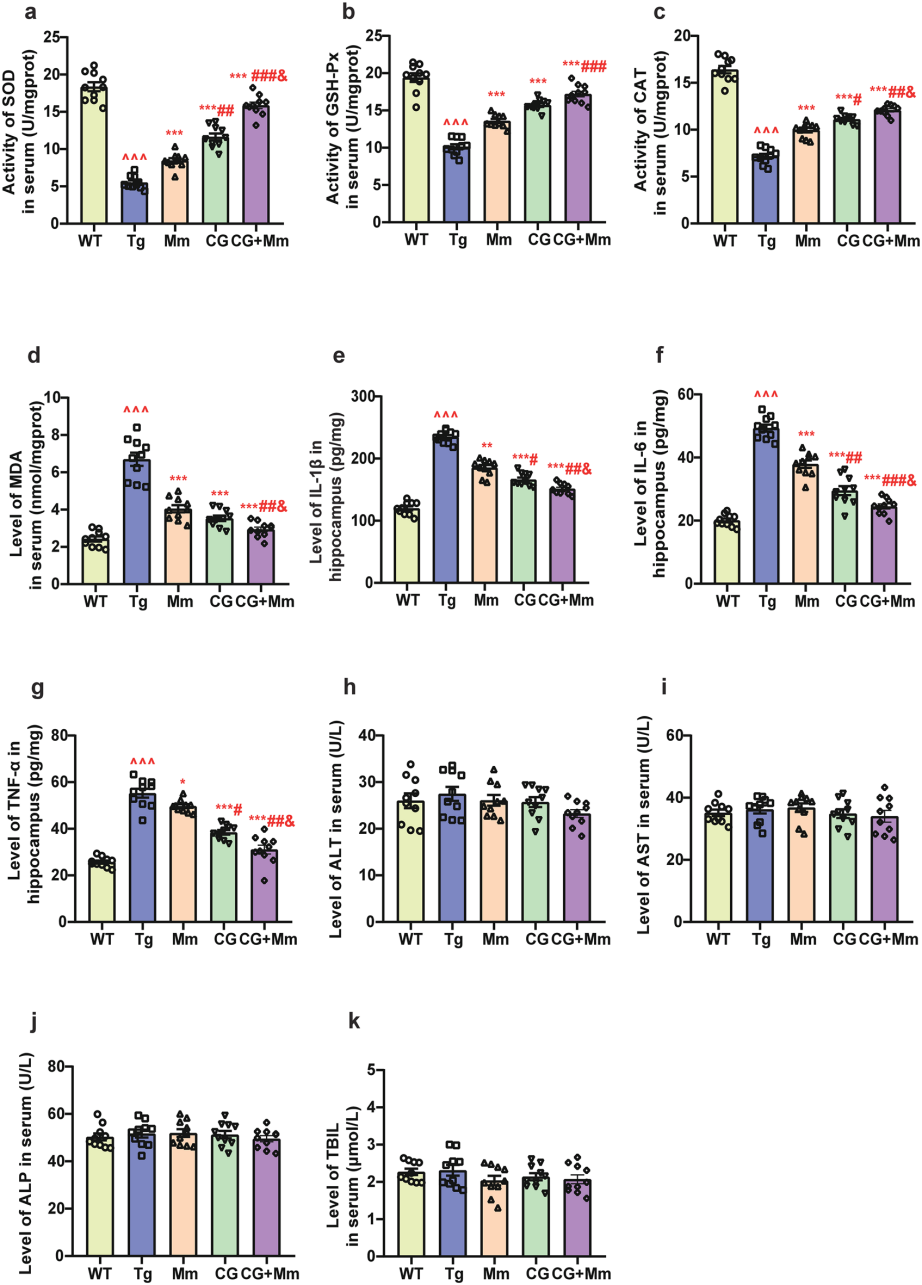
Effects of CG + Mm on the neuroinflammation, oxidative stress, and liver function in vivo. **a**–**c** The activities of SOD, GSH-Px, and CAT in serum, respectively. **d** The level of MDA in serum. **e**–**g** The levels of IL-1β, IL-6, and TNF-α in the hippocampus, respectively. **h**–**k** The levels of ALT, AST, ALP, and TBIL in serum, respectively. ^ *P* < 0.05, ^^ *P* < 0.01, ^^^ *P* < 0.01 vs. WT group; * *P* < 0.05, ** *P* < 0.01, *** *P* < 0.01 vs. Tg group; # *P* < 0.05, ## *P* < 0.001, ### *P* < 0.001, vs. Mm group, & *P* < 0.05, && *P* < 0.001, &&& *P* < 0.001, vs. CG group (n = 10)

The MDA contents in serum for Tg group were significantly higher compared to the WT group (*P* < 0.001, Fig. 5d). Moreover, the increase in MDA contents in serum was attenuated by CG + Mm administration. As shown in Fig. 5a–c, the activities of SOD, GSH-Px as well as the levels of CAT in the Tg group were significantly lower compared to the WT group in serum (*P* < 0.001), whereas CG + Mm treatment significantly alleviated the reduction of MDA (*P* < 0.001, Fig. 5d). In addition, oral administration of CG + Mm increased the levels of SOD, GSH-Px, and CAT in serum. Neuroinflammation is another pathological change of AD.6 In this study, we found that the level of inflammation in the hippocampus of APP/PS1 mice was signify-cantly increased (Levels of IL-1β, IL-6, TNF-α were significantly increased, *P* < 0.001, Fig. 5e–g), indicating the occurrence and development of neuroinflammation, while the therapeutic effect of Mm was limited. However, after Mm combined with CG, the development of inflammation was effectively controlled.

### Effects of CG + Mm on ALT, AST, ALP, and TBIL levels

To determine whether CG combined with Mm has potential hepatotoxicity, we measured the serum levels of ALT, AST, ALP, and TBIL (*P* > 0.05, Fig. 5h–k). The results showed that there was no significant difference between the five groups, which shows that CG combined with Mm is safe.

## Discussion

Current study, we observed that CG combined with Mm significantly improved the learning and memory abilities and autonomous activities of APP/PS1 mice, which are involved in reducing Aβ deposition (Fig. 4), improved synaptic plasticity, decreasing ROS production, inflammatory cytokines expression (Fig. 5) in AD mice brain. More and more evidence showed that abnormal production of Aβ is one of the important causes of AD [36, 37]. Extracellular Aβ oligomers trigger neuroinflammation and oxidative stress leading to cognitive decline with multiple types of neuronal damage in AD patients [38, 39]. Inhibition of Aβ production and deposition is considered to be a potential treatment strategy for AD. In this study, Mm showed a weak positive effect on reducing Aβ deposition. In comparison, there was an interesting result that CG had a better reducing Aβ deposition activity than Mm. CG contains a number of active ingredients, as one of the active ingredients in CG, ferulic acid reverses cognitive deficits and mitigates AD-like pathology by altering amyloido-genic β-secretase APP cleavage both in mutant APP-overexpressing neuron-like cells and transgenic mouse, hence, ferulic acid is considered as a β-secretase modulator [40, 41]. Rosmarinic acid suppresses AD development by reducing Aβ aggregation via increasing monoamine secretion [42]. Rhynchophylline suppresses soluble Aβ-induced impairment of spatial cognition function by inhibiting excessive activation of extrasynaptic NR2B-containing NMDA receptors [43]. Isorhynchophylline improves cognitive impairment in TgCRND8 mice via reducing Aβ generation and deposition, neuroinflammation through inhibiting the activation of JNK signaling pathway [44]. Thus, the mentioned ingredients of CG might serve as the key effectors in alleviating Aβ plaque burden and ameliorating cognitive decline during AD. Thus, we supposed that CG + Mm decreased Aβ production and accumulation by inhibiting expression of BACE1 and APP in APP/PS1 mouse brain, indicating that CG + Mm was involved in Aβ metabolism.

CG is a well-known Chinese patent medicine being sold in the current herbal market for years. To date, more than 100 biologically active compounds have been identified in CG. This herbal mixture is mainly used in treating headache and dizziness associated with cerebrovascular diseases in clinics [45]. Studies have shown that CG prevents aging mainly via suppression of oxidative stress response, such as decreasing NO and MDA levels, renewing activities of SOD, CAT, and GSH-Px, as well as decreasing AChE activity in the brain of d-gal-treated mice [45]. Previous researchers proved that paeoniflorin exerts neuroprotective effects by alleviating Aβ plaque burden, inhibiting astrocyte activation, and decreasing IL-1β and TNF-α expression in the brain of 5XFAD mice [46]. Tetrahydropalmatine prevents the neuroinflammation and memory impairment in the d-gal treated rats by decreasing the expression of nuclear factor κ (NF-κB) and glial fibrillary acidic protein [47]. Albiflorin ameliorates memory deficits in APP/PS1 mice by conferring synaptic protection and improving mitochondrial function, reducing Aβ deposition and reactive oxygen species in the brain [48]. These components make CG may have superior anti-inflammatory and anti-oxidative activities. Both AD patients and AD animals are suffering from chronic neuroinflammation and oxidative stress damage [8, 49]. Some researchers have suggested that Aβ disturbs the oxidative balance by causing mitochondrial dysfunction and lipid peroxidation to enhance oxidative stress [50]. In addition, glial cells can be over-activated by Aβ to release inflammatory factors to form a chronic neuroinflammatory environment in the brain [51]. Therefore, anti-inflammatory and anti-oxidative treatments are beneficial to AD. In this study, the activities of antioxidant enzymes (SOD, CAT, GSH-Px) in serum of AD mice in CG and Mm groups were significantly increased, as well as the inflammatory factors (IL-1β, IL-6, TNF-α) in hippocampus were significantly decreased, and this positive effect was amplified in the combined treatment. What's more, further studies are necessary to evaluate the underlying mechanism of CG and Mm at the cellular and molecular level.

Synaptic loss is correlated with memory and cognitive dysfunction in AD [52]. Synaptic plasticity is thought to be fundamental to learning and memory ability in the brain [53]. Thus, improving synaptic dysfunction might be a vital method to postpone the progression of the neurodegenerative disease. Synaptophysin is a pre-synaptic marker commonly used to detect the density and distribution of synapses. Synaptophysin loss is an early event of AD and is considered to be an important marker of synaptic changes [54]. PSD-95 is another important synaptic associated protein located in the post-synaptic region and plays an important role in synaptic plasticity [55]. In this study, Western blot analysis showed that the protein expression of synaptophysin and PSD-95 were significantly down-regulated in Tg group compared to the WT group. It is indicated that synaptic degeneration occurred in the mice of the Tg group. Our results showed that CG + Mm could restore the expression of synaptophysin and PSD-95, which implied that CG could resist the synaptic disruption. On the other hand, the combined group did not cause an increase in serum AST, ALT, ALP, and TBIL levels, indicating that there is no liver toxicity after long-term treatment.

Above all, our research suggests that a combination group could reach a better attenuate Alzheimer-like pathology effect than either of them. In our opinion, CG as a mixture of a few of active ingredients may enhance Mm anti-AD effect by reducing Aβ production and accumulation, neuroinflammation, oxidative damage and improving synaptic function through multiple signaling pathways and therefore could remedy AD cognitive impairment. Thus, the combination of CG with the Mm could be a better way to improve the treatment effects and life quality of AD patients.

## Conclusions

In conclusion, the present study has demonstrated that CG may enhance the Mm anti-AD effect of APP/PS1 mice. Based on these data, we hypothesized that the underlying mechanism of synergistic effect of CG and Mm was reducing of Aβ production via APP metabolic pathway and alleviating Aβ associated pathological events, oxidative stress injury, chronic neuroinflammation, and synaptic injury. These results provide a rationale for future clinical use of CG in anti-AD like pathological development which could play a role as complementary medicine. Moreover, further studies are necessary to evaluate the exact underlying mechanism of CG and CG + Mm at the cellular and molecular level.

## Data Availability

The research data generated from this study is included within the article.

## Abbreviations

AD: Alzheimer’s disease
CG: Cerebralcare Granule®
TCM: Traditional Chinese Medicine
Aβ: Amyloid-β
Mm: Memantine hydrochloride
HE: Hematoxylin–eosin
ALT: Alanine aminotransferase
AST: Aspartate aminotransferase
ALP: Alkaline phosphatase
TBIL: Total bilirubin
APP: Amyloid precursor protein
BACE1: β-Secretase
SYN: Synaptophysin
PSD95: Postsynaptic density 95
GAP43: Growth-associated protein 43
NMDA: Nmethyl-d-aspartate
WT: Wild type
Tg: APP/PS1 mice transgenic mice
MDA: Malondialdehyde
CAT: Catalase
GSH-Px: Glutathione peroxidase
SOD: Superoxide dismutase
IL-6: Interleukinn-6
IL-1β: Interleukin-1β
TNF-α: Tumour necrosis factor-α
EL: Escape latency
MWM: Morris water maze
OFT: Open field test
ANOVA: One-way analysis of variance

## References

[1] Cummings J, Lee G, Ritter A, Zhong K (2018). Alzheimer's disease drug development pipeline: 2018. Alzheimer's Dementia (New York, N Y).

[2] Lonnemann N, Hosseini S, Marchetti C, Skouras DB, Stefanoni D, D'Alessandro A (2020). The NLRP3 inflammasome inhibitor OLT1177 rescues cognitive impairment in a mouse model of Alzheimer's disease. Proc Natl Acad Sci U S A.

[3] Livingston G, Sommerlad A, Orgeta V, Costafreda SG, Huntley J, Ames D (2017). Dementia prevention, intervention, and care. Lancet.

[4] Li Z, Li H, Zhao CH, Lv C, Zhong CJ, Xin WF (2015). Protective effect of notoginsenoside R1 on an APP/PS1 mouse model of Alzheimer & aposs disease by up-regulating insulin degrading enzyme and inhibiting Aβ accumulation. CNS Neurol Disord Drug Targets.

[5] Pozueta J, Lefort R, Shelanski ML (2013). Synaptic changes in Alzheimer's disease and its models. Neuroscience.

[6] Zhu M, Wang X, Sun L, Marianne S, Erik H (2018). Can inflammation be resolved in Alzheimer's disease?. Ther Adv Neurol Disord.

[7] Blasko I, Veerhuis R, Stampfer-Kountchev M, Saurwein-Teissl M, Grubeck-Loebenstein B (2000). Costimulatory effects of interferon-γ and interleukin-1β or tumor necrosis factor α on the synthesis of Aβ1-40 and Aβ1-42 by human astrocytes. Neurobiol Dis.

[8] Ren P, Chen JW, Li BX, Zhang MZ, Yang B, Guo XS (2020). Nrf2 ablation promotes Alzheimer's disease-like pathology in APP/PS1 transgenic mice: the role of neuroinflammation and oxidative stress. Oxid Med Cell Longev.

[9] Gyorffy BA, Toth V, Torok G, Gulyassy P, Kovacs RA, Vadaszi H (2020). Synaptic mitochondrial dysfunction and septin accumulation are linked to complement-mediated synapse loss in an Alzheimer's disease animal model. Cell Mol Life Sci.

[10] Yang LL, Xue Y, Wei JC, Dai Q, Li P (2021). Integrating metabolomic data with machine learning approach for discovery of Q-markers from Jinqi Jiangtang preparation against type 2 diabetes. Chin Med.

[11] Wang DM, Liu L, Li SQ, Wang CY (2018). Effects of paeoniflorin on neurobehavior, oxidative stress, brain insulin signaling, and synaptic alterations in intracerebroventricular streptozotocin-induced cognitive impairment in mice. Physiol Behav.

[12] Mori T, Koyama N, Yokoo T, Segawa T, Maeda M, Sawmiller D (2020). Gallic acid is a dual α/β-secretase modulator that reverses cognitive impairment and remediates pathology in Alzheimer mice. J Biol Chem.

[13] Thingore C, Kshirsagar V, Juvekar A (2021). Amelioration of oxidative stress and neuroinflammation in lipopolysaccharide-induced memory impairment using Rosmarinic acid in mice. Metab Brain Dis.

[14] Lee TK, Kang IJ, Kim B, Sim HJ, Kim DW, Ahn JH (2020). Experimental pretreatment with chlorogenic acid prevents transient ischemia-induced cognitive decline and neuronal damage in the hippocampus through anti-oxidative and anti-inflammatory effects. Molecules.

[15] Meng GL, Meng XL, Ma XY, Zhang GP, Hu XL, Jin AP (2018). Application of ferulic acid for Alzheimer's disease: combination of text mining and experimental validation. Front Neuroinform.

[16] Li XW, Tong L, Li YF, Sun GX, Yang DL, Herry S (2016). Simultaneous determination of seven alkaloids in rat plasma by UFLC-MS/MS and its application to a pharmacokinetic study after oral administration of Cerebralcare Granule. J Chromatogr B Analyt Technol Biomed Life.

[17] Wang XY, Ma XH, Li W, Chu Y, Guo JH, Li SM (2013). Simultaneous determination of five phenolic components and paeoniflorin in rat plasma by liquid chromatography-tandem mass spectrometry and pharmacokinetic study after oral administration of Cerebralcare granule (R). J Pharm Biomed Anal.

[18] Lin YL, Yang XG, Lu M, Zheng WJ, Zhang J, Zhuang HQ (2013). Herbal compound triptolide synergistically enhanced antitumor activity of vasostatin120-180. Anticancer Drugs.

[19] Wang XN, Zhao HF, Liu LM, Niu P, Zhai C, Li JN (2020). Hejie Zhitong prescription promotes sleep and inhibits nociceptive transmission-associated neurotransmitter activity in a rodent migraine model. Chin Med.

[20] Xia JF, Rong L, Sawakami T, Inagaki Y, Song PP, Hasegawa K (2018). Shufeng Jiedu Capsule and its active ingredients induce apoptosis, inhibit migration and invasion, and enhances doxorubicin therapeutic efficacy in hepatocellular carcinoma. Biomed Pharmacother.

[21] Zheng F, Zhao YY, Li X, Tang Q, Wu JJ, Wu WY (2019). The repression and reciprocal interaction of DNA methyltransferase 1 and specificity protein 1 contributes to the inhibition of MET expression by the combination of Chinese herbal medicine FZKA decoction and erlotinib. J Ethnopharmacol.

[22] Zhang XQ, Ding YW, Chen JJ, Xiao X, Zhang W, Zhou L (2020). Xiaoaiping injection enhances paclitaxel efficacy in ovarian cancer via pregnane X receptor and its downstream molecules. J Ethnopharmacol.

[23] Li MF, Xie L, Li Y, Liu J, Nie GN, Yang HY (2020). Synergistic effect of Huyang Yangkun Formula and embryonic stem cells on 4-vinylcyclohexene diepoxide induced premature ovarian insufficiency in mice. Chin Med.

[24] Olivares D, Deshpande VK, Shi Y, Lahiri DK, Greig NH, Rogers JT (2012). N-methyl D-aspartate (NMDA) receptor antagonists and memantine treatment for Alzheimer's disease, vascular dementia and Parkinson's disease. Curr Alzheimer Res.

[25] Monika J, Tamara Z, Aveli N, Margarita M, Allan B, Max Z (2019). Effects of the drug combination memantine and melatonin on impaired memory and brain neuronal deficits in an amyloid-predominant mouse model of Alzheimer's disease. J Pharm Pharmacol.

[26] Zhang JH, Yu LJ, Hui Y, Zhen H, Su J, Ling C (2018). Huatuo Zaizao pill ameliorates cognitive impairment of APP/PS1 transgenic mice by improving synaptic plasticity and reducing Aβ deposition. BMC Complement Altern Med.

[27] Yang M, Sun JH, Lu ZQ, Chen GT, Guan SH, Liu X (2008). Phytochemical analysis of traditional Chinese medicine using liquid chromatography coupled with mass spectrometry. J Chromatogr.

[28] Fernand VE, Dinh DT, Washington SJ, Fakayode SO, Losso JN, van Ravenswaay RO (2008). Determination of pharmacologically active compounds in root extracts of *Cassia alata* L. by use of high performance liquid chromatography. Talanta.

[29] Ding B, Zhou T, Fan GR, Hong ZY, Wu YT (2007). Qualitative and quantitative determination of ten alkaloids in traditional Chinese medicine *Corydalis yanhusuo* WT Wang by LC-MS/MS and LC-DAD. J Pharm Biomed Anal.

[30] Chen L, Qi J, Chang YX, Zhu DN, Yu BY (2009). Identification and determination of the major constituents in Traditional Chinese Medicinal formula Danggui-Shaoyao-San by HPLC-DAD-ESI-MS/MS. J Pharm Biomed Anal.

[31] Zhang HY, Hu P, Luo GA, Liang QL, Wang YL, Yan SK (2006). Screening and identification of multi-component in Qingkailing injection using combination of liquid chromatography/time-of-flight mass spectrometry and liquid chromatography/ion trap mass spectrometry. Anal Chim Acta.

[32] Long QH, Wu YG, He LL, Ding L, Tan AH, Shi HY (2021). Suan-Zao-Ren Decoction ameliorates synaptic plasticity through inhibition of the Aβ deposition and JAK2/STAT3 signaling pathway in AD model of APP/PS1 transgenic mice. Chin Med.

[33] Hardy J, Selkoe DJ (2002). Medicine—the amyloid hypothesis of Alzheimer's disease: progress and problems on the road to therapeutics. Science.

[34] Kastanenka KV, Hou SS, Shakerdge N, Logan R, Feng D, Wegmann S (2017). Optogenetic restoration of disrupted slow oscillations halts amyloid deposition and restores calcium homeostasis in an animal model of Alzheimer's disease. PLoS ONE.

[35] Fol R, Braudeau J, Ludewig S, Abel T, Weyer SW, Roederer JP (2016). Viral gene transfer of APPs alpha rescues synaptic failure in an Alzheimer's disease mouse model. Acta Neuropathol.

[36] Desgranges B, Baron JC, de la Sayette V, Petit-Taboue MC, Benali K, Landeau B (1998). The neural substrates of memory systems impairment in Alzheimer's disease - A PET study of resting brain glucose utilization. Brain.

[37] Xia DY, Huang X, Bi CF, Mao LL, Peng LJ (2017). PGC-1α or FNDC5 Is involved in modulating the effects of Aβ142 oligomers on suppressing the expression of BDNF, a beneficial factor for inhibiting neuronal apoptosis, Aβ deposition and cognitive decline of APP/PS1 Tg mice. Front Aging Neurosci.

[38] Cavallucci V, D’Amelio M, Cecconi F (2012). Aβ toxicity in Alzheimer's disease. Mol Neurobiol.

[39] Reiserer RS, Harrison FE, Syverud DC, McDonald MP (2007). Impaired spatial learning in the APP(Swe)+PSEN1 Delta E9 bigenic mouse model of Alzheimer's disease. Genes Brain Behav.

[40] Mori T, Koyama N, Guillot-Sestier MV, Tan J, Town T (2013). Ferulic acid is a nutraceutical β-secretase modulator that improves behavioral impairment and alzheimer-like pathology in transgenic mice. PLoS ONE.

[41] Mori T, Koyama N, Tan J, Segawa T, Maeda M, Town T (2019). Combined treatment with the phenolics (-)-epigallocatechin-3-gallate and ferulic acid improves cognition and reduces Alzheimer-like pathology in mice. J Biol Chem.

[42] Hase T, Shishido S, Yamamoto S, Yamashita R, Nukima H, Taira S (2019). Rosmarinic acid suppresses Alzheimer's disease development by reducing amyloid β aggregation by increasing monoamine secretion. Sci Rep.

[43] Yang Y, Ji WG, Zhu ZR, Wu YL, Zhang ZY, Qu SC (2018). Rhynchophylline suppresses soluble Aβ 1–42 -induced impairment of spatial cognition function via inhibiting excessive activation of extrasynaptic NR2B-containing NMDA receptors. Neuropharmacology.

[44] Li HQ, Ip SP, Yuan QJ, Zheng GQ, Tsim KKW, Dong TTX (2019). Isorhynchophylline ameliorates cognitive impairment via modulating amyloid pathology, tau hyperphosphorylation and neuroinflammation: studies in a transgenic mouse model of Alzheimer's disease. Brain Behav Immun.

[45] Qu Z, Yang HG, Zhang JZ, Huo LQ, Chen H, Li YM (2016). Cerebralcare Granule(A (R)), a Chinese Herb compound preparation, attenuates d-galactose induced memory impairment in mice. Neurochem Res.

[46] Kong YY, Peng QJ, Lv N, Yuan J, Deng ZR, Liang XL (2020). Paeoniflorin exerts neuroprotective effects in a transgenic mouse model of Alzheimer's disease via activation of adenosine A(1) receptor. Neurosci Lett.

[47] Qu Z, Zhang JZ, Yang HG, Huo LQ, Gao J, Chen H (2016). Protective effect of tetrahydropalmatine against D-galactose induced memory impairment in rat. Physiol Behav.

[48] Xu YJ, Mei Y, Shi XQ, Zhang YF, Wang XY, Guan L (2019). Albiflorin ameliorates memory deficits in APP/PS1 transgenic mice via ameliorating mitochondrial dysfunction. Brain Res.

[49] Nayuta N, Haruo H, Daisuke H, Hirokuni H, Tomohiko S, Soichiro S (2017). Oxidative stress and inflammation are associated with physical frailty in patients with Alzheimer's disease. Geriatr Gerontol Int.

[50] Zhou WW, Lu S, Su YJ, Xue D, Yu SL, Wang SW (2014). Decreasing oxidative stress and neuroinflammation with a multifunctional peptide rescues memory deficits in mice with Alzheimer disease. Free Radic Biol Med.

[51] Cheng YW, Chang CC, Chang TS, Li HH, Hung HC, Liu GY (2019). Aβ stimulates microglial activation through antizyme-dependent downregulation of ornithine decarboxylase. J Cell Physiol.

[52] Selkoe DJ, Schenk D (2003). Alzheimer's disease: Molecular understanding predicts amyloid-based therapeutics. Annu Rev Pharmacol Toxicol.

[53] Losonczy A, Makara JK, Magee JC (2008). Compartmentalized dendritic plasticity and input feature storage in neurons. Nature.

[54] Masliah E, Mallory M, Alford M, DeTeresa R, Hansen LA, McKeel DW (2001). Altered expression of synaptic proteins occurs early during progression of Alzheimer's disease. Neurology.

[55] El-Hussein AE, Schnell E, Chetkovich DM, Nicoll RA, Bredt DS (2000). PSD-95 involvement in maturation of excitatory synapses. Science.

